# Human essential hypertension: no significant association of polygenic risk scores with antihypertensive drug responses

**DOI:** 10.1038/s41598-020-68878-3

**Published:** 2020-07-20

**Authors:** Heini Sánez Tähtisalo, Sanni Ruotsalainen, Nina Mars, Kimmo Porthan, Lasse Oikarinen, Juha Virolainen, Frej Fyhrquist, Samuli Ripatti, Kimmo K. Kontula, Timo P. Hiltunen

**Affiliations:** 1 Department of Medicine, University of Helsinki and Helsinki University Hospital, Helsinki, Finland; 2 Research Program for Clinical and Molecular Metabolism, Faculty of Medicine, University of Helsinki, Helsinki, Finland; 3 Institute for Molecular Medicine Finland, Helsinki Institute of Life Sciences, University of Helsinki, Helsinki, Finland; 4 Division of Cardiology, Heart and Lung Center, University of Helsinki and Helsinki, University Central Hospital, Helsinki, Finland; 5 Department of Medicine, University of Helsinki and Minerva Foundation Institute for Medical Research, Helsinki, Finland; 6 Minerva Foundation Institute for Medical Research, Helsinki, Finland; 7 Department of Public Health, University of Helsinki, Helsinki, Finland

**Keywords:** Cardiovascular genetics, Genetics research, Hypertension, Cardiovascular genetics, Hypertension, Hypertension, Molecular medicine, Pharmacogenetics

## Abstract

Polygenic risk scores (PRSs) for essential hypertension, calculated from > 900 genomic loci, were recently found to explain a significant fraction of hypertension heritability and complications. To investigate whether variation of hypertension PRS also captures variation of antihypertensive drug responsiveness, we calculated two different PRSs for both systolic and diastolic blood pressure: one based on the top 793 independent hypertension-associated single nucleotide polymorphisms and another based on over 1 million genome-wide variants. Using our pharmacogenomic GENRES study comprising four different antihypertensive monotherapies (n ~ 200 for all drugs), we identified a weak, but (after Bonferroni correction) statistically nonsignificant association of higher genome-wide PRSs with weaker response to a diuretic. In addition, we noticed a correlation between high genome-wide PRS and electrocardiographic left ventricular hypertrophy. Finally, using data of the Finnish arm of the LIFE study (n = 346), we found that PRSs for systolic blood pressure were slightly higher in patients with drug-resistant hypertension than in those with drug-controlled hypertension (*p* = 0.03, not significant after Bonferroni correction). In conclusion, our results indicate that patients with elevated hypertension PRSs may be predisposed to difficult-to-control hypertension and complications thereof. No general association between a high PRS and less efficient drug responsiveness was noticed.

## Introduction

Elevated blood pressure (BP) has emerged as the leading risk factor for global disease burden, with a prevalence of 24% in males and 20% in females and totalling 1.1 billion affected adults worldwide^1^. Hypertension has been estimated to account for approximately 9.4 million deaths annually^2^. The insidious nature of hypertension is underscored by its high prevalence, its mostly asymptomatic nature, and slow progress in achievement of targets of treatment, calling for need of fundamental transformation in attempts of hypertension control^3^.

There is much hope that better understanding of the underlying genetic mechanisms would result in more precise ways of screening, diagnostic classification and drug treatment of hypertension. Although major progress has been encountered in studies on pathophysiology and individualized treatment of monogenic forms of hypertension^4–6^, the clinical usefulness of genomic techniques in patients with essential hypertension has remained limited. Genome-wide association studies (GWASs) have revealed up to 900 genetic loci associated with elevated BP^7–10^, but each locus mostly accounts for a very small degree (typically, 0.2 mmHg) of BP variation. Pharmacogenomic approaches have provided an alternative strategy to identify genetic markers of therapeutic responsiveness. Although large collaborative studies have reported promising genetic variants related to antihypertensive drug responses^11–17^, their ultimate clinical impact remains to be explored.

Calculation of polygenic risk scores (PRSs) from GWAS results has been shown to provide a promising technique for risk assessment of a number of complex diseases, including coronary artery disease, atrial fibrillation, type 2 diabetes, inflammatory bowel disease and breast cancer^18^. The recent extension of BP-associated genomic markers showed that a PRS calculated across 901 independent genetic loci identified was associated with approximately 10 mmHg BP difference between the top and bottom quintiles of the PRS distribution^10^. In addition, PRS was associated with increased risk of myocardial infarction and stroke, with odds ratios of about 1.5 comparing top and bottom deciles of the PRS distribution^10^.

In order to investigate whether variation of PRS based on all hypertension associated single-nucleotide polymorphisms (SNPs) also signals variation to antihypertensive drug responsiveness, we used our pharmacogenomic GENRES (Genetics of Drug Responsiveness in Essential Hypertension) Study as a primary platform^13,19^. In GENRES, the antihypertensive effects of four different drug classes (a diuretic, a beta blocker, a calcium channel blocker and an angiotensin receptor antagonist) were studied in a placebo-controlled, rotational fashion, and genotyping was performed for the DNA samples, permitting calculation of PRSs for hypertension. We decided to replicate the corresponding data on beta blocker and angiotensin receptor antagonist responses using DNA samples and BP data from the Finnish arm of the LIFE (Losartan Intervention for Endpoint Reduction in Hypertension) Study^20^. Accordingly, our data set provides a tool to investigate whether PRSs for BP also signifies antihypertensive drug responsiveness and whether any relation noted shows drug specificity.

## Results

### Patient characteristics

Baseline characteristics for both GENRES and LIFE study subjects included in the analyses of BP responses are summarised in Table 1. Most noticeable differences between the GENRES and LIFE study populations were higher age, inclusion of females, and higher placebo systolic blood pressure (SBP) levels in the LIFE Study.

**Table 1:** Characteristics of the study subjects included in the analyses of blood pressure responses.

Parameters	GENRES	LIFE	
		Atenolol	Losartan
n	203–207^a^	201	200
Age (years)	50.6 ± 6.4	64.0 ± 6.5	63.9 ± 5.9
Men (%)	100	52	48
Body mass index (kg m^−2^)	26.7 ± 2.7	27.2 ± 3.6	27.7 ± 3.4
Current smoker (%)	16	10	12
Creatinine (µmol/l)	86 ± 13	80 ± 13	82 ± 17
Potassium (mmol/l)	4.4 ± 0.3	4.3 ± 0.3	4.2 ± 0.3
Urate (µmol/l)	372 ± 64	327 ± 66	336 ± 70
Glucose (mmol/l)	5.4 ± 0.6	5.5 ± 1.1	5.7 ± 1.5
Total cholesterol (mmol/l)	5.6 ± 0.9	6.1 ± 0.9	5.9 ± 1.0
**Blood pressure levels on placebo**			
Ambulatory SBP (mmHg)	135 ± 10	NA	NA
Ambulatory DBP (mmHg)	93 ± 6	NA	NA
Office SBP (mmHg)	152 ± 13	166 ± 13	166 ± 12
Office DBP (mmHg)	100 ± 7	98 ± 6	97 ± 6
**Blood pressure responses**^**b**^			
Amlodipine			
ΔSBP (mmHg)	− 7.4 ± 7.2	NA	NA
ΔDBP (mmHg)	− 4.9 ± 4.0	NA	NA
Beta blocker^c^			
ΔSBP (mmHg)	− 11.1 ± 6.2	− 21.4 ± 12.8	NA
ΔDBP (mmHg)	− 8.3 ± 4.2	− 12.8 ± 6.6	NA
Hydrochlorothiazide			
ΔSBP (mmHg)	− 4.8 ± 6.3	NA	NA
ΔDBP (mmHg)	− 1.7 ± 4.1	NA	NA
Losartan			
ΔSBP (mmHg)	− 9.1 ± 6.7	NA	− 20.8 ± 12.2
ΔDBP (mmHg)	− 6.1 ± 4.7	NA	− 10.9 ± 7.3
**Genetic risk scores**			
Top_PRS_SBP_	2.10 ± 1.87	1.87 ± 1.86	2.02 ± 1.93
Top_PRS_DBP_	0.63 ± 1.04	0.36 ± 1.13	0.41 ± 1.13
GW_PRS_SBP_	0.53 ± 0.22	0.45 ± 0.24	0.46 ± 0.23
GW_PRS_DBP_	0.57 ± 0.23	0.45 ± 0.24	0.46 ± 0.24

Values are presented as mean ± s.d., unless otherwise stated.

*Δ* change, *DBP* diastolic blood pressure, *GW* genome-wide, *PRS* polygenic risk score, *SBP* systolic blood pressure.

^a^Depending on the drug (see “Methods” section).

^b^Blood pressure responses are from 24-h ambulatory recordings for GENRES and from office measurements for LIFE.

^c^Bisoprolol in GENRES, atenolol in LIFE.

### Polygenic risk scores in GENRES and LIFE

**Figure 1:**
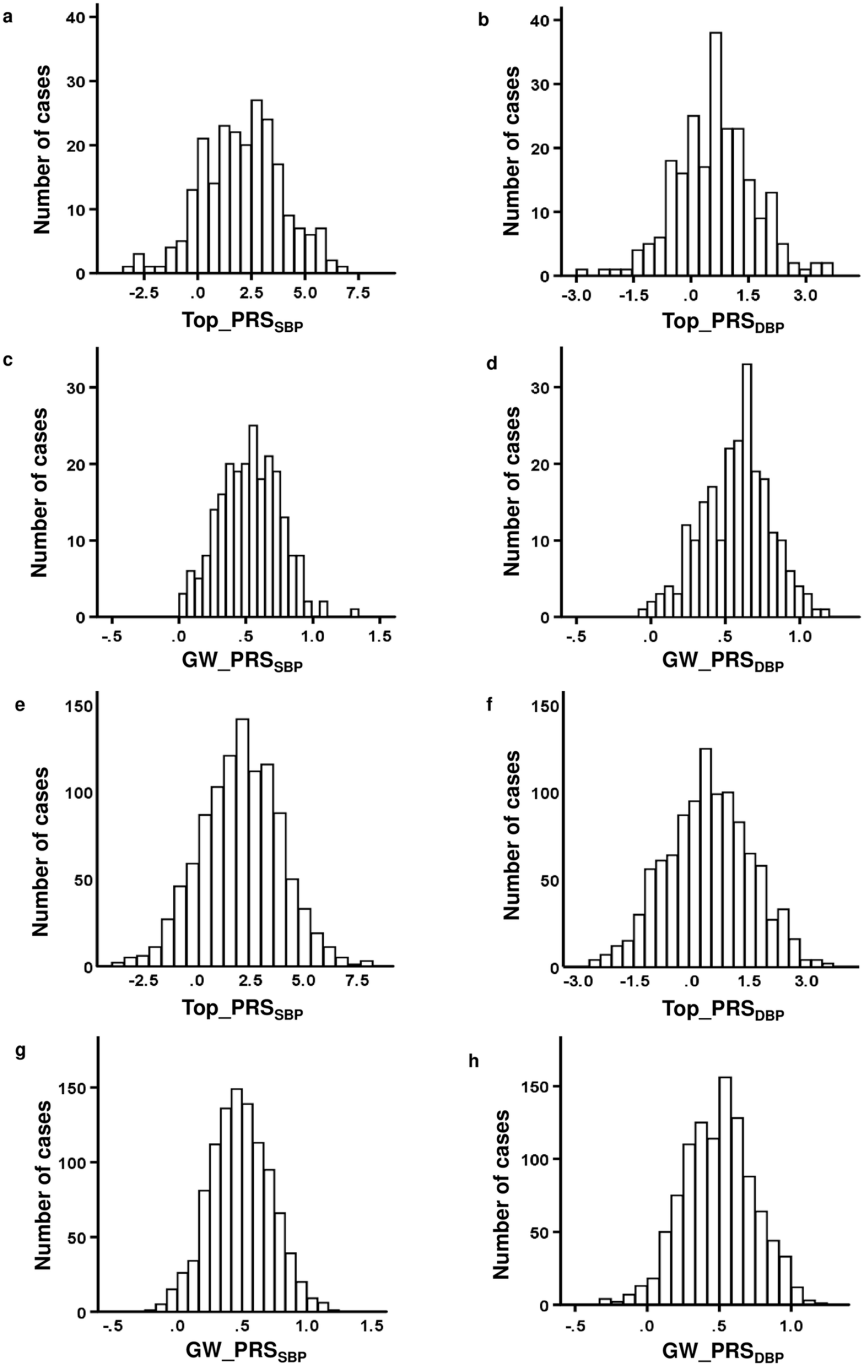
Distributions of Top_PRSs and GW_PRSs in GENRES (**a**–**d**) and LIFE (**e**–**h**). All PRSs are unitless and expressed as relative values.

We calculated two different PRSs for both SBP and diastolic blood pressure (DBP): one based on the top 793 independent BP-associated SNPs (Top_PRS) and another based on 1 million genome-wide variants (GW_PRS). All four PRSs, including Top_PRS_SBP_, Top_PRS_DBP_, GW_PRS_SBP_ and GW_PRS_DBP_, were normally distributed in both the GENRES and the LIFE subjects (Fig. 1).

We validated our PRS estimates by calculation of their reciprocal correlations with placebo BP levels. In both patient cohorts, Top_PRS_SBP_ was correlated with Top_PRS_DBP_ (GENRES: r = 0.84; LIFE: r = 0.83), and GW_PRS_SBP_ was correlated with GW_PRS_DBP_ (GENRES: r = 0.76; LIFE: r = 0.76) (Supplementary Fig. S1 online). Furthermore, Top_PRS_SBP_ and GW_PRS_SBP_ were also correlated with each other (GENRES: r = 0.60; LIFE: r = 0.60), and likewise Top_PRS_DBP_ and GW_PRS_DBP_ were correlated (GENRES: r = 0.50; LIFE: r = 0.53) (Supplementary Fig. S2 online).

In GENRES there were positive correlations between all PRSs and placebo BP levels (r values 0.16–0.22, *p *values 0.001–0.02; Supplementary Table S1 online). In LIFE, these correlations were also positive and most reached statistical significance (Supplementary Table S1 online).

### PRSs and left ventricular hypertrophy in GENRES

As a further tool to validate our PRS data, we took advantage of the electrocardiographic (ECG) and echocardiographic studies of all GENRES patients. GW_PRS_SBP_ correlated significantly with ECG-estimated QRS area (*p* = 0.0004) and nonsignificantly with left ventricular mass index (LVMI) as estimated by echocardiography (*p* = 0.07), and with Sokolow-Lyon voltage (*p* = 0.04) and Cornell voltage product (*p* = 0.02) in ECG recordings (Table 2). GW_PRS_DBP_ was positively, but statistically nonsignificantly, correlated with QRS area (*p* = 0.05). Finally, in analyses of Top_PRSs and measures of LVH, Top_PRS_SBP_ correlated nonsignificantly with QRS area (*p* = 0.04). Collectively, our data suggest that elevated PRSs increase risk of left ventricular hypertrophy in hypertensive patients.

**Table 2:** Correlation of GW_PRSs (a) and Top_PRSs (b) with measures of left ventricular hypertrophy (LVH) during placebo periods in GENRES.

LVH measures	n	GW_PRS_SBP_		GW_PRS_DBP_	
		Std beta	*p*	Std beta	*p*
**a**					
Left ventricular mass index	221	0.12	0.07	− 0.01	0.91
Sokolow-Lyon voltage	227	0.13	0.04	0.08	0.21
Cornell voltage product	226	0.16	0.02	0.01	0.93
QRS area sum	227	0.24	0.0004	0.13	0.051

LVH measures	n	Top_PRS_SBP_		Top_PRS_DBP_	
		Std beta	*p*	Std beta	*p*
**b**					
Left ventricular mass index	221	0.03	0.63	− 0.01	0.89
Sokolow-Lyon voltage	227	0.02	0.71	0.04	0.57
Cornell voltage product	226	0.13	0.06	0.08	0.23
QRS area sum	227	0.14	0.04	0.11	0.10

Linear regression with covariates listed in “Methods” section was used. Bonferroni-corrected *p *values < 0.0018 were considered statistically significant.

### PRSs and antihypertensive drug responses

In order to practically elucidate the possible associations between patient-specific PRSs and antihypertensive drug responses, we carried out two types of mathematical analyses. First, correlations between PRSs and covariate-adjusted BP responses in the two studies are summarised in Table 3. No statistically significant associations, meeting the Bonferroni-corrected *p *value limit 0.003, were observed. The strongest observed correlations were those of antihypertensive responses to hydrochlorothiazide with genome-wide PRSs in GENRES, suggesting that higher PRSs were associated with weaker BP responses to this diuretic.

**Table 3:** Correlations between PRSs and covariate-adjusted blood pressure responses in GENRES (a) and LIFE (b).

	Blood pressure responses							
	Amlodipine		Bisoprolol		Hydrochlorothiazide		Losartan	
	n = 205		n = 207		n = 206		n = 203	
	r	*p*	r	*p*	r	*p*	r	*p*
**a. GENRES**								
Top_PRS_SBP_	0.05	0.48	− 0.10	0.17	0.05	0.51	0.02	0.77
Top_PRS_DBP_	− 0.01	0.84	− 0.02	0.83	0.09	0.20	− 0.01	0.88
GW_PRS_SBP_	0.05	0.44	− 0.03	0.63	0.16*	0.03	0.06	0.38
GW_PRS_DBP_	0.08	0.24	− 0.04	0.59	0.18*	0.01	0.06	0.43

	Blood pressure responses							
	Atenolol		Losartan					
	n = 201		n = 200					
	r	*p*	r	*p*				
**b. LIFE**								
Top_PRS_SBP_	0.006	0.89	0.01	0.77				
Top_PRS_DBP_	0.007	0.89	− 0.06	0.20				
GW_PRS_SBP_	− 0.03	0.48	0.03	0.57				
GW_PRS_DBP_	0.03	0.48	− 0.03	0.53				

PRSs for SBP were correlated with SBP responses and PRSs for DBP were correlated with DBP responses. Pearson correlation test was used for these analyses; a positive r-value indicates a weaker BP response with increasing PRS value.

*r values 0.16/0.18 correspond to 0.92/0.69 mmHg changes in BP responses per 1 SD changes in GW_PRS_SBP_/GW_PRS_DBP_.

**Figure 2:**
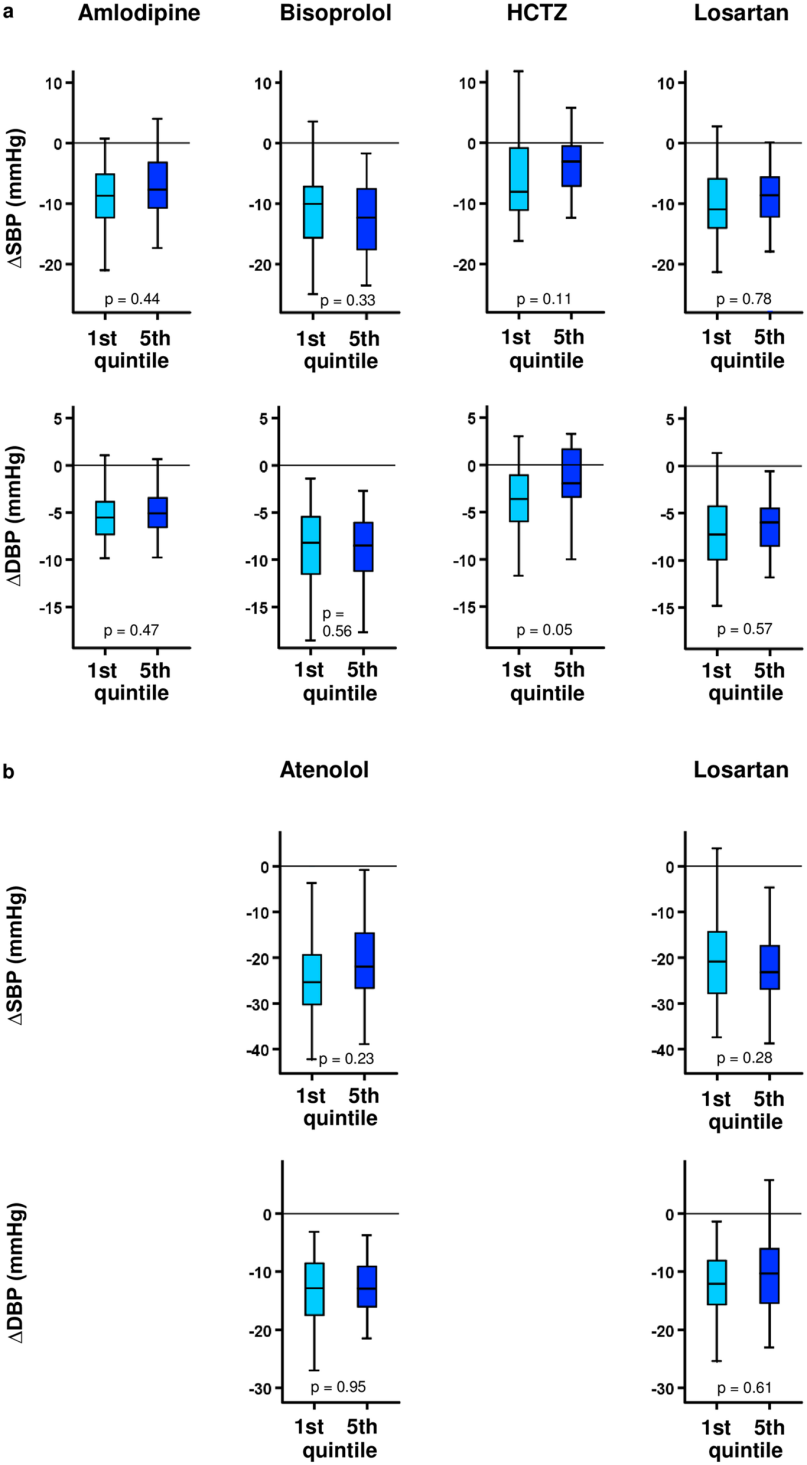
Covariate-adjusted blood pressure responses by the lowest/highest genome-wide PRS quintiles in GENRES (**a**) and LIFE (**b**). Box-and-whisker plots are presented. *p *values are from Student’s *t* test when comparing the lowest and the highest PRS quintiles. *HCTZ* hydrochlorothiazide.

Second, in order to further characterize the drug responsiveness of the patients with the lowest and highest PRSs, an additional analysis was carried out in the first and fifth PRS quintiles. There were suggestive but statistically nonsignificant associations between the extreme GW_PRS quintiles and systolic (*p* = 0.11) or diastolic (*p* = 0.05) BP responses to hydrochlorothiazide, while no evidence was found for similar associations for the other three drugs (Fig. 2a). A partial replication study was conducted in the LIFE Study: bisoprolol responses in GENRES were compared to atenolol responses in LIFE, and losartan responses in both studies were compared; no significant correlations between the PRSs and drug responses were noticed (Fig. 2b).

Due to the observed suggestive associations of genome-wide PRSs with BP responses to hydrochlorothiazide (Table 3), their predictive performance for good BP response was further evaluated using receiver operating characteristic (ROC) analysis. The threshold for good response to hydrochlorothiazide was set at covariate-adjusted BP change better than − 0.5 s.d. (corresponding to − 8.0/− 3.8 mmHg SBP/DBP responses). The area under ROC curve for SBP response, predicted by GW_PRS_SBP_, was 0.64 (*p* = 0.0009; the highest Youden index 0.25 with a sensitivity of 0.61 and a specificity of 0.63), and the corresponding results for DBP response, predicted by GW_PRS_DBP_, was 0.63 (*p* = 0.002; the highest Youden index 0.24 with a sensitivity of 0.48 and a specificity of 0.77) (Supplementary Fig. S3 online). Regardless of the *p* values, the area under curve and the highest Youden index values are low and indicate poor predictive value of the genome-wide PRSs for practical prediction of BP responses to hydrochlorothiazide.

### PRSs and drug treatment-resistant hypertension

**Figure 3:**
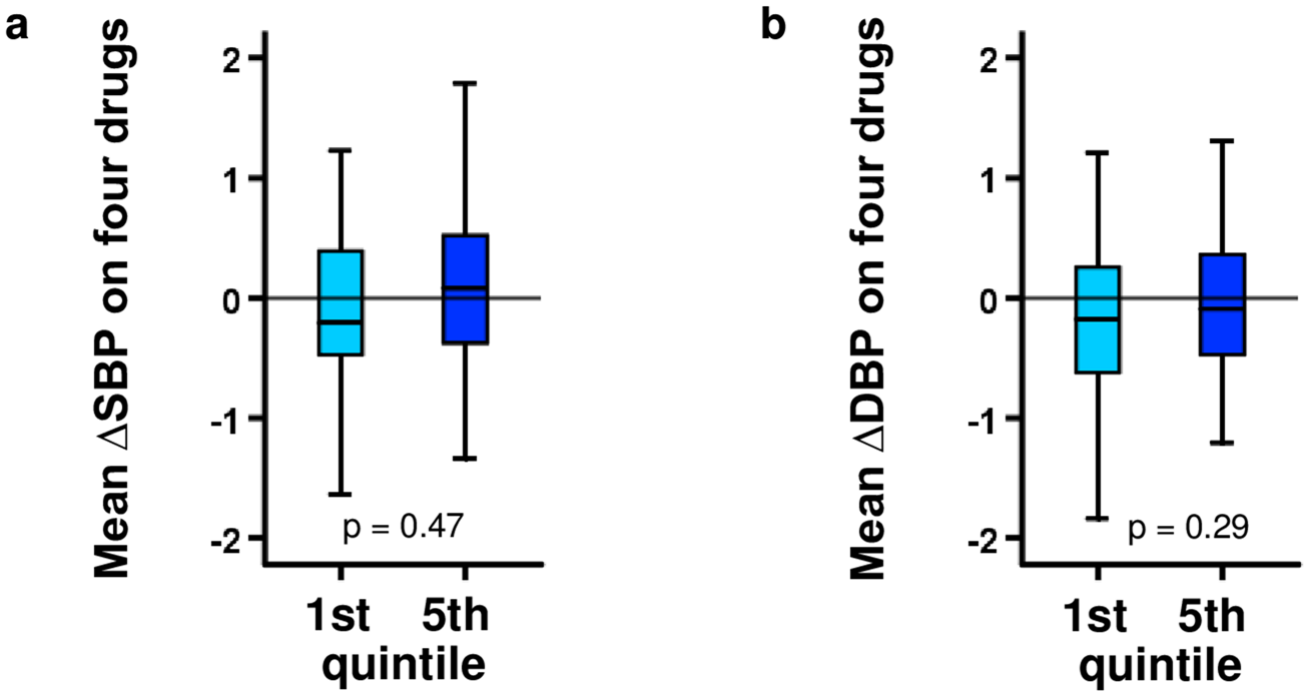
Mean covariate-adjusted SBP (**a**) and DBP (**b**) change on the four study drugs in the lowest/highest GW_PRS quintiles in GENRES. The BP changes are shown as s.d. units; box-and-whisker plots are presented. *p *values are from Student’s *t* test when comparing the lowest and the highest PRS quintiles.

In order to obtain a surrogate index of drug sensitivity and drug resistance in the GENRES Study, we calculated a mean standardized response rate using data for all four antihypertensive drugs used in the study (see “Methods” section). There was only a weak and statistically nonsignificant correlation of the genome-wide risk scores with the mean integrated BP responses in GENRES: GW_PRS_SBP_ vs SBP response (r = 0.11, *p* = 0.12), GW_PRS_DBP_ versus DBP response (r = 0.10, *p* = 0.15). On the same line, when the subjects were grouped into quintiles on the basis of their PRSs, no differences between the extreme PRS quintiles emerged (Fig. 3).

**Figure 4:**
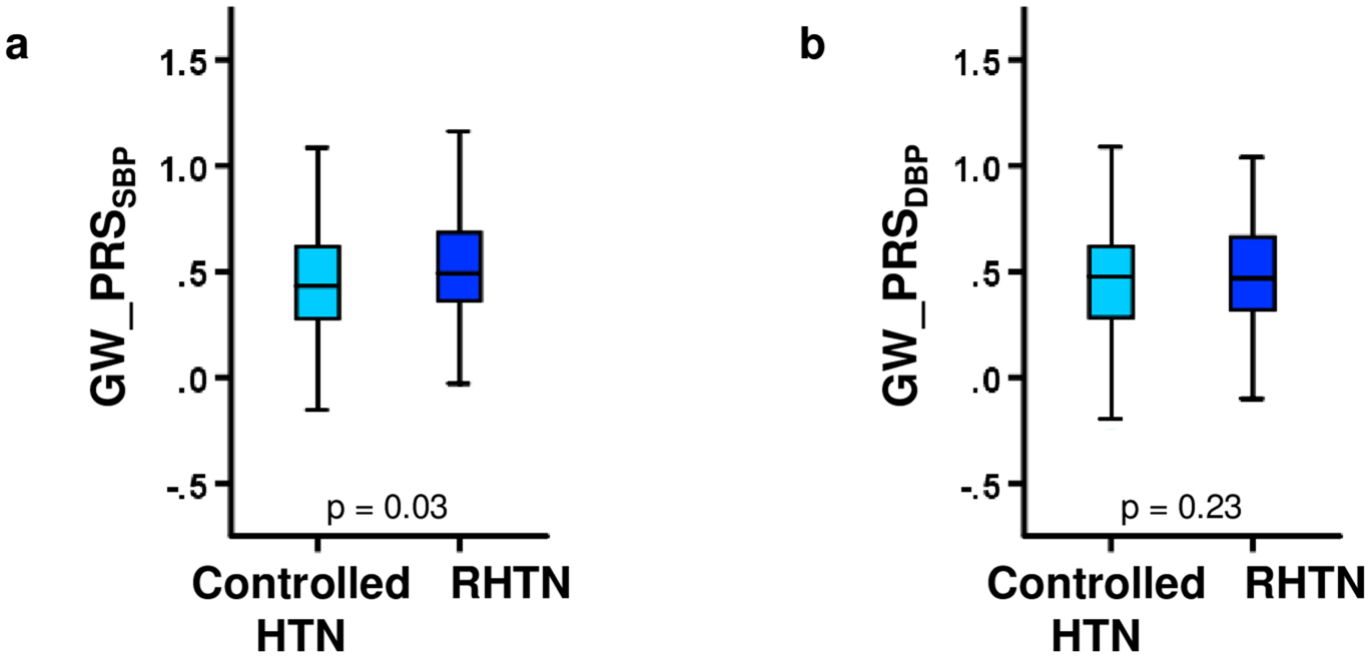
Comparison of genome-wide polygenic risk scores for SBP (**a**) and DBP (**b**) between subjects with treatment-resistant hypertension (RHTN) and controlled hypertension (HTN) in LIFE at 2 years of the study. Box-and-whisker plots are presented. *p *values are from Student’s *t* test when comparing subjects with controlled and treatment-resistant hypertension. Both PRSs are unitless and expressed as relative values.

We next explored whether there is evidence for association of high PRS with resistance to antihypertensive therapy in the LIFE Study. In LIFE, 177 subjects were judged to have controlled hypertension and 169 subjects to have treatment-resistant hypertension at 2 years of the study. Their baseline characteristics are summarised in Supplementary Table S2 online. GW_PRS_SBP_ was found to be slightly, but (after Bonferroni correction) statistically nonsignificantly higher in the group of treatment-resistant hypertension than in controlled hypertension (*p* = 0.03 in *t* test and *p* = 0.02 in covariate-adjusted linear regression), while no difference was noted in the corresponding analysis of GW_PRS_DBP_ (Fig. 4). Similar, statistically nonsignificant results were revealed for Top_PRS_SBP_ (*p* = 0.05 in *t* test and *p* = 0.05 in covariate-adjusted linear regression) and no difference was observed in comparison of the Top_PRS_DBP_ of controlled and treatment-resistant hypertensive patients of the LIFE study (Supplementary Table S2 online).

### PRSs and cardiovascular endpoints in the LIFE Study

A total of 70 subjects in the LIFE Study experienced a primary composite endpoint (cardiovascular death, stroke, myocardial infarction), while 977 subjects did not. Their baseline characteristics and the results from Cox regression analysis of the association between the PRSs and the occurrence of the primary composite endpoint are summarised in Supplementary Table S3 online. In endpoint analyses, only a nonsignificant association between GW_PRS_SBP_ and occurrence of the endpoint was seen (*p* = 0.08 in Cox regression analysis, Supplementary Table S3 online).

## Discussion

We investigated whether variation of PRSs based on all presently known hypertension-associated SNPs signals variation on BP responses to four commonly used classes of antihypertensive drugs. We found some, but considering the multiple comparisons, statistically nonsignificant evidence for higher PRSs associating with less efficient BP response to a thiazide diuretic, but no support for similar associations between PRSs and effects of three other classes of antihypertensive drugs. Furthermore, we observed a significant relation between high GW_PRS_SBP_ and an index of LVH, as well as a suggestive but statistically nonsignificant association between high PRS values and occurrence of drug-resistant hypertension. The present study appears to be the first on genomic risk scores in prediction of antihypertensive drug effects, as previous studies have been concentrated on hypertension per se^10,18,21^, or on complications of hypertension^22,23^.

In order to identify tools for optimal use of polygenic risk scores for future drug response studies of human hypertension, we used four different genomic risk scores for our analyses: two based on 793 SNPs associated with systolic or diastolic BP, and two genome-wide risk scores taking into account genomic variation at over a million of loci. The conventional way of calculating PRSs has been to calculate them from a subset of independent, associated genetic markers based on GWAS summary statistics (applying LD pruning and *p *value threshold), corresponding to the Top_PRSs of the current study. This approach has advantages in terms of computational simplicity and has been used to predict genetic liability across a broad phenotypic spectrum^24^. In the current study, we used a single *p *value threshold of < 0.05 but it is also possible to apply more relaxed *p *value thresholds for the calculation of the Top_PRSs, which might improve their performance. However, finding the optimal threshold for genome-wide prediction would need a large training set independent of the original GWAS and was outside of the scope of this study. Recently, Bayesian methods to generate a SNP set for PRS by calculating the posterior effect sizes from GWAS summary statistics conditioned by an LD information from a reference panel have been increasingly used^24,25^. The main advantage from this method compared to conventional LD pruning and *p *value threshold-based method is that it includes SNPs across a broad range of *p *values and may thus account for possible hidden signals retrieved by omitting pruning^25^. This prompted us to use also the GW_PRSs in our calculations.

Furthermore, we took advantage of use of two meticulously phenotyped samples of hypertensive patients, originating from the Finnish population. We obtained qualification for the use of these cohorts by showing that higher PRSs were correlated with higher baseline (placebo) BP levels in both GENRES and LIFE (Supplementary Table S1 online).

When analysing associations between PRSs and antihypertensive responses of four drug classes, it was intriguing to notice that any indication for possible relation between elevated GW_PRS and weaker BP response was observed only for a diuretic (Table 3 and Fig. 2). Although the extent of this relation did not reach statistical significance after the conservative Bonferroni correction on this occasion, we take our finding as an interesting hint toward further investigations. Accordingly, whether this preliminary finding indicates that the genomic loci relevant to PRS calculation reflect mechanisms that are especially associated with volume expansion awaits for additional studies. Regarding the Top_PRSs, they did not display any association with BP responses to the diuretic. This is probably related to their only moderate correlation with the GW-PRSs (r values ~ 0.6 and r^2^ values ~ 0.36; see also Supplementary Fig. S2 online) and may be a sign of BP-associated loci that fall below genome-wide significance.

Evidence for the assumption that a high hypertension PRS is an indication for more intensive drug therapy comes from two other lines of the present study. First, we observed certain correlations between PRSs and measures of LVH. Echocardiographic evidence of LVH was available from the GENRES group, indicating a trend toward correlation between LVMI and the GW_PRS_SBP_ (Table 2). In addition, three electrocardiographic LVH indices showed significant or suggestive correlations with the patients’ PRSs (Table 2).

Second, some evidence favouring use of PRS values for predictive purposes comes from analyses of resistant patients. Resistant hypertension is often defined as the lack of attainment of a target BP level despite the use of at least three different antihypertensive drugs, of which at least one should include a diuretic^26^. Resistant hypertension is a distinctly ominous feature, associating with increased risk of complications, including myocardial infarction, congestive heart failure, stroke and kidney failure. We were able to directly analyse the possible relation of drug-resistant hypertension to PRS values in the LIFE cohort. Indeed, GW_PRS_SBP_ was nominally associated (*p* = 0.03) with drug-resistant hypertension in LIFE (Fig. 4). Our end-point analysis data in LIFE (Supplementary Table S3 online) did not reveal statistically significant associations, possibly due to the facts that the number of endpoints among the Finnish LIFE patients was limited and because these patients were already selected according to their elevated risk. However, our data are not in disagreement with the end-point data of Evangelou et al.^10^, originally providing the platform of hypertension PRS estimation. It is also of note that in GENRES we noticed a trend toward less efficient BP lowering in patients with high PRSs when we used a composite response index taking into account all individual responses to the four drug classes (a diuretic, beta-blocker, calcium channel antagonist, angiotensin receptor antagonist) used. These data on drug resistance justify careful replication studies coming from other pharmacogenomic studies of human hypertension. In summary, we have generated data supporting the assumption that hypertension PRSs may have predictive role in identification of patients requiring more intensive drug treatment to reach BP targets and to avoid complications of hypertension. However, since most of these findings did not reach statistical significance when performance of multiple comparisons was considered, these aspects require further studies in other populations.

Our study has some important limitations. Although in its design the GENRES Study is an almost ideal for pharmacogenomic studies, its sample size is limited and it consists of males only; in addition, there is no follow-up data for the GENRES patients. The Top_PRSs were calculated with a single and strict *p *value threshold of < 0.05 and yielded associations less significant than those obtained with the GW_PRSs. It is, however, possible that Top_PRSs based on more relaxed *p *value thresholds could perform better. Due to the design of the LIFE Study, data on thiazide and calcium channel antagonist could not be replicated in it. In addition, only subjects on monotherapy at 2 months’ visit were included in the BP response analyses of the LIFE Study, which excludes many nonresponders and reduces the power of the analyses. It should also be emphasized that 24-h ambulatory BP data were used in GENRES while office BP measurements took place in LIFE.

In conclusion, our data indicate that patients with elevated hypertension PRSs are predisposed to difficult-to-control hypertension and complications thereof. Whether a high PRS indeed signals less efficient responsiveness to thiazide diuretics awaits additional investigations in other, and possibly larger, clinical studies.

## Methods

### Patients

The general design of the GENRES Study has been described previously^19^. In brief, it is a randomized, double-blind, placebo-controlled, rotational study using four different antihypertensive monotherapies. The study subjects were 35–60 year old Finnish men with moderate hypertension. The study protocol included a 4-week initial wash-out placebo period, followed by 4-week drug monotherapy periods (hydrochlorothiazide 25 mg, bisoprolol 5 mg, losartan 50 mg, amlodipine 5 mg), separated by 4-week placebo periods (Supplementary Fig. S4 online). Measurement of office BP and 24-h ambulatory BP recording were carried out after each drug and placebo period. For the present study, we selected the patients with imputed genotype data and ambulatory BP response data for at least one drug (205 for amlodipine, 207 for bisoprolol, 206 for hydrochlorothiazide, and 203 for losartan). The clinical part of the study was carried out in accordance with the Declaration of Helsinki and Guidelines for Good Clinical Practice (1996). The study was approved by the Ethics Committee of Helsinki University Hospital and the National Agency for Medicines of Finland. All screened subjects gave a signed informed consent prior to study activities.

The LIFE study is an international, randomized, double-blind study originally aimed at evaluating the long-term treatment effects of losartan compared with atenolol in 9,193 hypertensive patients with signs of LVH^27^. Patients were randomly assigned to treatment with losartan (50 mg daily) or atenolol (50 mg daily) after a 2-week wash-out placebo period. Hydrochlorothiazide 12.5 mg daily was then added to the treatment if target BP (< 140/90 mmHg) was not achieved. Later treatment escalations included increase of the study drug dose to 100 mg, increase of hydrochlorothiazide dose to 25 mg and addition of other antihypertensive drugs (Supplementary Fig. S4 online). A pharmacogenetic substudy was done in Scandinavia, including 1,146 Finnish patients whose DNA samples were available for GWAS^20^. Of this group, a total of 401 patients on monotherapy at 2 months’ visit (losartan, n = 200; atenolol, n = 201) were selected for analysis of BP responses in the current study. The numbers of subjects included for analysis of treatment-resistant hypertension and for cardiovascular endpoints were 346 and 1,047, respectively (see below). The main treatment protocol of the LIFE study and the protocol of the genetics substudy were approved by local ethical committees and done in accordance with the Declaration of Helsinki. All participants gave a written informed consent before study.

### Electrocardiographic and echocardiographic studies in the GENRES Study

A digital standard resting 12-lead ECG was recorded at the end of all eight GENRES study periods (Marquette MAC 5,000 electrocardiograph, GE Marquette Medical Systems, Milwaukee, WI). QT Guard 1.3 software (GE Marquette Medical Systems) produced automatically digital median QRS-T complexes for all 12 leads. Electrocardiographic evidence for LVH was explored using three different methods: calculation of the QRS area (mean integral value of the QRS complexes of all leads), estimation of Sokolow-Lyon voltage (defined as S_V1_ + R_V5_ or R_V6_, whichever was greater) or calculation of Cornell voltage product, defined as (S_V3_ + R_aVL_) × QRS duration.

Transthoracic echocardiography was performed at the end of the first placebo period in the GENRES Study. All echocardiographic measurements were averaged from five cardiac cycles, and were available for 221 (96.9%) subjects. Left ventricular mass in grams was calculated with an anatomically validated formula: 0.8 × [1.04 × ((interventricular septal thickness + left ventricular end-diastolic diameter + posterior wall thickness)^3 ^ – left ventricular end-diastolic diameter^3^)] + 0.6. LVMI was indexed to body surface area.

### Treatment-resistant hypertension in the LIFE Study

Treatment-resistant hypertension status was defined at the 2 years’ visit, which allowed enough time for BP medication titration and reduced the number of exclusions (due to experiencing study outcomes or censoring). Treatment-resistant hypertension was defined as SBP ≥ 140 mmHg or DBP ≥ 90 mmHg and the use of at least three different antihypertensive drugs. Controlled hypertension was defined as SBP < 140 mmHg and DBP < 90 mmHg with a maximum of three drugs.

Before exclusions, 387 subjects could be classified to treatment-resistant or controlled hypertension. After exclusions [loss from follow-up (n = 3), morbid obesity (body mass index > 40 kg/m^2^, n = 6), macroalbuminuria (> 300 mg/l, n = 3), unsuccessful genotyping (n = 26), and occurrence of a primary composite endpoint before year 2 (stroke, n = 3)], 169 subjects with treatment-resistant hypertension, and 177 with controlled hypertension remained in the analysis.

### Cardiovascular endpoints in the LIFE Study

For the analysis of cardiovascular endpoints, the primary composite endpoint (cardiovascular mortality, stroke, and myocardial infarction) of the LIFE Study was used^27^.

After exclusions [morbid obesity (body mass index > 40 kg/m^2^, n = 14), macroalbuminuria (> 300 mg/l, n = 12), first degree relativeness (n = 6), and unsuccessful genotyping (n = 67)], the total amount of subjects included in the analyses was 1,047; the primary composite endpoint occurred in 70 (6.9%) of them.

### Genotyping and imputation

The genotyping methods and quality control steps for GENRES and LIFE have been described in detail before^13,20^. The DNA samples were genotyped at the Institute for Molecular Medicine Finland (FIMM, Helsinki, Finland) using the Illumina HumanOmniExpress BeadChip (Illumina, San Diego, CA, USA). Genotype data and reference genome builds were lifted over to build version 38 (GRCh38/hg38) following the protocol described (dx.doi.org/10.17504/protocols.io.nqtddwn). In sample-wise quality control, individuals with high genotype missingness (> 5%) and excess heterozygosity (± 4SD) were removed; there were no subjects of non-Finnish ancestry. In variant-wise quality control prior imputation variants with high missingness (> 2%), low HWE *p* value (< 1 × 10^–6^) and minor allele count < 3 were removed. Phasing and imputation of the genotypes were done utilizing a Finnish population-specific reference panel (SISu v3) of 3,775 high-coverage whole-genome sequences as described (dx.doi.org/10.17504/protocols.io.nmndc5e).

### Calculation of individual polygenic risk scores

We calculated two different patient-specific PRSs for both SBP and DBP: one based on the top 793 independent (LD-pruned) hypertension-associated SNPs (Top_PRS) listed in Supplementary Table S4 online, and another genome-wide PRS (GW_PRS). Information on the presently known genomic loci associated with hypertension per se was obtained from the study of Evangelou et al.^10^.

For calculation of Top_PRS for each subject, we selected only those 793 SNPs that were associated (*p* < 0.05) with either SBP or DBP, omitting SNPs that were associated with only pulse pressure^10^. The allelic weights were directly acquired from the data of Evangelou et al.^10^ by request.

In order to calculate the GW_PRSs for each individual we used the PRS-CS method^24^, which is a Bayesian method to infer posterior effect sizes for variants using summary statistics from GWAS and an external linkage disequilibrium (LD) reference panel.

In calculation of GW_PRSs, we used 1000G EUR^28^ as LD reference panel and limited our calculation for variants from Hapmap3 phase 3^29^ totalling 1,085,815 for SBP and 1,085,696 variants for DBP.

### Statistics

The statistical analyses were run using SPSS version 22.0 (IBM SPSS Statistics, Armonk, NY). All analysed BP values in the GENRES study were based on ambulatory 24-h recordings. The mean BP level of all (up to four) placebo periods was used as the baseline level. BP responses to antihypertensive drugs were calculated as BP after 4 weeks’ drug treatment minus baseline BP. In the LIFE study, BP levels were derived from office measurements. BP responses were calculated as BP after 2 months’ drug treatment minus BP after the 2-week wash-out placebo period. Normality of data was assessed using skewness of the distributions.

The associations of PRSs with BP responses were analysed using BP response residuals generated with stepwise linear regression. Covariates used for BP residual calculations in GENRES were chosen from the following parameters in a drug-specific fashion using stepwise regression (*p* < 0.10 as the inclusion criterion): the corresponding mean BP level on all placebo periods, age, earlier use of antihypertensive medication, current smoking, body mass index, and daily urinary sodium excretion and serum creatinine after the first placebo period^13^. The distributions of the BP response residuals did not deviate significantly from normal distribution, as judged by evaluation of skewness and Kolmogorov–Smirnov test (smallest *p *value was 0.08). The distributions of the BP response residuals are shown in Supplementary Fig S5 online. In LIFE, the following covariates were used in addition to the corresponding BP level on placebo for all BP responses: sex, age and body mass index. Covariates included are listed in a cohort- and drug-specific way in Supplementary Tables S5 and S6 online.

Pearson correlation test was used to analyse the association between PRSs and BP response residuals. To further describe the associations between PRSs and BP responses, both study populations were divided into PRS quintiles. The lowest and the highest quintiles were then compared with Student’s *t* test.

To analyse the association of PRSs with the mean BP response to the four drugs in the GENRES study, each ambulatory BP response to the study drug was covariate-adjusted and standardized (mean = 0, SD = 1) after which the means of the standardized SBP and DBP responses to the four study drugs were calculated. The mean standardized responses were then analysed in a way similar to the monotherapy BP responses.

The predictive performance of PRSs for good BP response was evaluated using ROC analysis with SPSS program (version 22.0, IBM SPSS Statistics, Armonk, NY).

The associations between PRSs and LVH measures were analysed with linear regression. In addition to the analysed PRS, covariates were included in the model using stepwise regression (*p* < 0.10 as an inclusion condition). The covariates tested were age, body mass index, height and body surface area. Of these covariates, age (but none of the body size-related parameters) was included in analyses of LVMI (*p *values ranging from 0.007 to 0.008 with the various PRSs). In the analyses of the ECG measures of LVH, body mass index was the only additional covariate that was included in the models: its *p *values ranged from 9 × 10^–6^ to 0.0001 for Sokolow-Lyon voltage, from 0.01 to 0.07 for Cornell voltage product, and from 0.003 to 0.002 for QRS area. Normalized values were used for QRS area because of its non-normal distribution. Electrocardiographic recordings were available from up to four placebo periods and the means of them were used in the analyses.

In LIFE, the PRSs between subjects with treatment-resistant hypertension and controlled hypertension were compared using Student’s *t* test. Linear regression analysis (SPSS General Linear Model) was run to verify the results using sex, current smoking, treatment arm, estimated glomerular filtration rate, and body mass index as covariates.

Cox regression was used to analyse the association of PRSs with the occurrence of the primary composite endpoint using the following covariates: sex, current smoking, diabetes, earlier cardiovascular disease, treatment arm, and age.

The drug response study included multiple comparisons due to several primary target variables (systolic and diastolic BP responses to four drug treatments, and two separate PRSs for each response). For these analyses, the Bonferroni-corrected *p* value limit for statistical significance was set at 0.003 (= 0.05/16). This approach can be considered very conservative, since the systolic and diastolic BP responses to each drug correlate highly (r values > 0.8) as do the top-SNP and genome-wide PRSs (r values ~ 0.6). For the analysis of the secondary target variables, the Bonferroni-corrected *p* value was calculated considering the following analyses: four different LVH measures and four PRSs, two mean BP responses (systolic and diastolic) to the four study drugs in GENRES and two PRSs for both responses, treatment-resistant hypertension in LIFE and four PRSs, cardiovascular endpoints in LIFE and four PRSs. This gives a Bonferroni-corrected *p *value limit of 0.0018 (0.05/(4 × 4 + 2 × 2 + 4 + 4)) for statistical significance.

To assess the statistical power of our primary correlation analysis, we calculated the power for three different correlation coefficients: 0.1, 0.18 and 0.4. We used n = 207, corresponding to the GENRES data set sample size, and the Bonferroni-corrected *p *value threshold of 0.003. The corresponding powers were: 0.06, 0.35 and 0.999.

## Data availability

All relevant data are in the manuscript and supplementary materials.

## Supplementary information

Supplementary Information 1.

Supplementary Information 2.
