# Supplementary material for: Human essential hypertension: no significant association of polygenic risk scores with antihypertensive drug responses
Source: Sci Rep. 2020 Jul 20;10:11940. doi: 10.1038/s41598-020-68878-3 (PMC7371738; doi:10.1038/s41598-020-68878-3)

Human essential hypertension: No significant association of polygenic risk scores with antihypertensive drug responses

Heini Sánez Tähtisalo[#], Sanni Ruotsalainen[#], Nina Mars, Kimmo Porthan, Lasse Oikarinen, Juha Virolainen, Frej Fyhrquist, Samuli Ripatti, Kimmo K. Kontula, Timo P. Hiltunen

SUPPLEMENTARY MATERIALS

Supplementary Tables S1-3 and S5-6 and Supplementary Figures S1-5

[#] These authors contributed equally to this work

SUPPLEMENTARY TABLES

Supplementary Table S1. Correlations between PRSs and placebo BP levels in GENRES and LIFE.

	Placebo blood pressure levels							
	GENRES (n = 228)				LIFE (n = 1047)			
	SBP		DBP		SBP		DBP	
	r	p	r	p	r	p	r	p
Top_PRS_{SBP}	0.20	0.003			0.02	0.5		
Top_PRS_{DBP}			0.16	0.02			0.09	0.003
GW_PRS_{SBP}	0.22	0.001			0.10	0.002		
GW_PRS_{DBP}			0.16	0.02			0.12	0.00005

Supplementary Table S2. Characteristics of the LIFE study subjects included in the analyses of treatment-resistant hypertension.

	Treatment-resistant	Controlled hypertension
n	169	177
Age (years)	64.3 ± 6.3	62.3 ± 5.9
Men (%)	43	54
Body mass index (kgm^{-2})	28.7 ± 4.1	27.5 ± 3.4
Current smoker (%)	8	12
Creatinine (μmol/l)	80 ± 14	82 ± 12
Potassium (mmol/l)	4.1 ± 0.3	4.2 ± 0.3
Urate (μmol/l)	340 ± 81	338 ± 69
Glucose (mmol/l)	5.9 ± 1.6	5.5 ± 1.2
Total cholesterol (mmol/l)	6.0 ± 1.0	5.9 ± 0.9
Blood pressure levels on placebo		
SBP (mmHg)	180 ±13	164 ± 13
DBP (mmHg)	101 ± 9	98 ± 7
Blood pressure responses at the 2-year visit		
ΔSBP (mmHg)	-23.0 ± 16.7	-32.4 ± 12.2
ΔDBP (mmHg)	-14.4 ± 7.9	-16.8 ± 6.9
Genetic risk scores		
Top_PRS_{SBP}	2.12 ± 1.59	1.74 ± 2.03
Top_PRS_{DBP}	0.35 ± 0.96	0.26 ± 1.17
GW_PRS_{SBP}	0.51 ± 0.24	0.45 ± 0.25
GW_PRS_{DBP}	0.49 ± 0.24	0.46 ± 0.26

Values are presented as mean ± s.d., unless otherwise stated. PRSs are unitless and expressed as relative values.
Δ, change; DBP, diastolic blood pressure; GW, genome-wide; PRS, polygenic risk score; SBP, systolic blood pressure.

Supplementary Table S3. Characteristics of the LIFE study subjects included in endpoint analysis, and the results from Cox regression analysis of the association between the PRSs and the occurrence of the primary composite endpoint.

	**Primary composite endpoint**	**Controls**
n	70	977
Age at randomization (years)	66.3 ± 6.7	63.8 ± 6.2
Men (%)	56	48
Body mass index (kgm^{-2})	28.2 ± 4.0	27.8 ± 3.6
Current smoker (%)	16	10
Creatinine (µmol/l)	85 ± 16	81 ± 17
Potassium (mmol/l)	4.2 ± 0.4	4.2 ± 0.3
Urate (µmol/l)	333 ± 56	337 ± 72
Glucose (mmol/l)	5.9 ± 2.0	5.6 ± 1.4
Total cholesterol (mmol/l)	6.1 ± 1.0	6.0 ± 1.0
Blood pressure levels on placebo		
SBP (mmHg)	176 ± 14	172 ± 14
DBP (mmHg)	98 ± 9	99 ± 8
Blood pressure responses at the 2-year visit		
ΔSBP (mmHg)	-24.2 ± 16.0	-23.5 ± 16.5
ΔDBP (mmHg)	-14.7 ± 8.9	-13.2 ± 8.1
Genetic risk scores*		
Top_PRS_{SBP}	2.14 ± 2.03	2.03 ± 1.86
Top_PRS_{DBP}	0.30 ± 1.23	0.43 ± 1.10
GW_PRS_{SBP}	0.52 ± 0.24	0.48 ± 0.23
GW_PRS_{DBP}	0.50 ± 0.24	0.49 ± 0.25

Values are presented as mean ± s.d., unless otherwise stated. PRSs are unitless and expressed as relative values.

*Results from Cox regression analysis of the association between the PRSs and the occurrence of the primary composite endpoint: Top_PRS_{SBP}, $p = 0.55$; Top_PRS_{DBP}, $p = 0.66$; GW_PRS_{SBP}, $p = 0.08$; GW_PRS_{DBP}, $p = 0.33$.

Δ, change; DBP, diastolic blood pressure; GW, genome-wide; PRS, polygenic risk score; SBP, systolic blood pressure.

Supplementary Table S5. Covariates included in blood pressure residual calculations in GENRES.

	Blood pressure responses															
	**Amlodipine**				**Bisoprolol**				**Hydrochlorothiazide**				**Losartan**			
	Systolic		Diastolic		Systolic		Diastolic		Systolic		Diastolic		Systolic		Diastolic	
	Std beta	p	Std beta	p	Std beta	p	Std beta	p	Std beta	p	Std beta	p	Std beta	p	Std beta	p
Mean systolic BP on placebo*	-0.44	$2×10^{-12}$	-	-	-0.19	0.006	-	-	-0.28	$4×10^{-5}$	-	-	-0.21	0.003	-	-
Mean diastolic BP on placebo	-	-	-0.39	$1×10^{-9}$	-	-	-0.26	$1×10^{-4}$	-	-	-0.26	$2×10^{-4}$	-	-	-0.25	$3×10^{-4}$
Age*	-0.22	$2×10^{-4}$	-0.26	$4×10^{-5}$	-	-	-	-	-0.19	$4×10^{-3}$	-0.12	0.08	-	-	-	-
Earlier antihypertensive use (no=0, yes=1)	-	-	-0.13	0.03	-	-	-	-	-	-	-	-	-	-	-	-
Smoking (no=0, yes=1)	-	-	-	-	0.12	0.08	-	-	-	-	-	-	-	-	-	-
BMI	-	-	0.11	0.07	-	-	-	-	-	-	-	-	-	-	-	-
dU-sodium*	0.25	$3×10^{-5}$	0.16	0.01	-	-	-	-	0.12	0.07	0.12	0.08	-	-	-	-
Serum creatinine	-	-	-	-	-	-	-	-	-	-	-	-	-0.15	0.03	-0.17	0.01

A negative standardized beta indicates a more negative BP change (= better antihypertensive response) with increasing covariate value. Normalized values were used for non-normally distributed variables.

Std beta, standardized beta; BMI, body mass index; BP, blood pressure; dU, daily urinary excretion.

Supplementary Table S6. Covariates included in blood pressure residual calculations in LIFE.

	Blood pressure responses							
	Atenolol				Losartan			
	Systolic		Diastolic		Systolic		Diastolic	
	Std beta	p	Std beta	p	Std beta	p	Std beta	p
Mean systolic BP on placebo	-0.53	4×10^{-14}	-	-	-0.57	2×10^{-16}	-	-
Mean diastolic BP on placebo	-	-	-0.55	2×10^{-15}	-	-	-0.57	5×10^{-17}
Age	0.04	0.52	-0.19	0.004	0.07	0.28	-0.26	5×10^{-5}
BMI	0.01	0.84	-0.002	0.97	0.11	0.06	-0.03	0.59
Sex (male=0, female=1)	0.02	0.81	-0.03	0.64	-0.03	0.63	-0.01	0.90

A negative standardized beta indicates a more negative BP change (= better antihypertensive response) with increasing covariate value.
Std beta, standardized beta; BMI, body mass index; BP, blood pressure.

SUPPLEMENTARY FIGURES

Supplementary Figure S1. Correlations between systolic and diastolic PRSs in GENRES (A) and LIFE (B). PRSs are unitless and expressed as relative values.

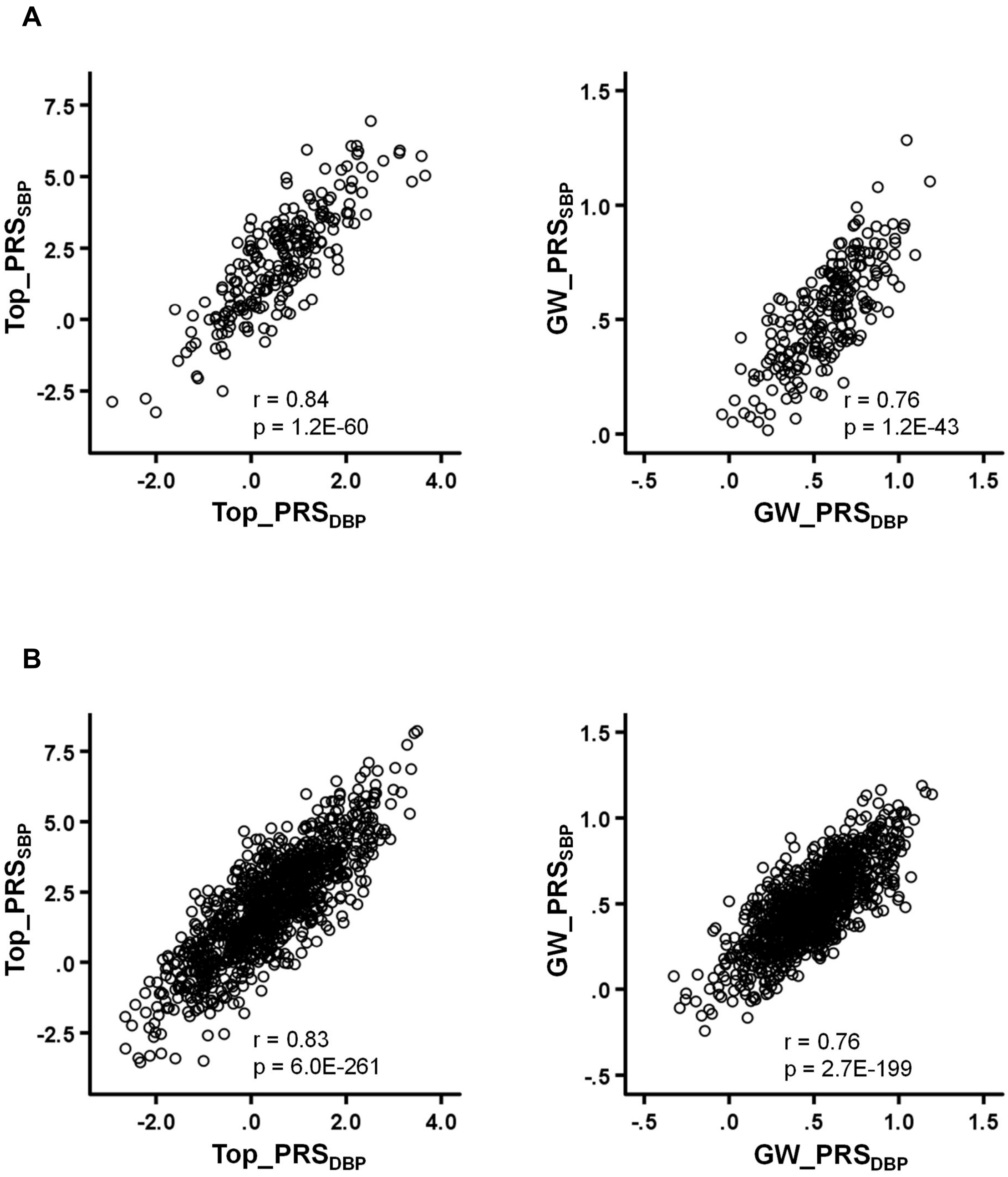

Supplementary Figure S2. Correlations between Top_PRSs and GW_PRSs in GENRES (A) and LIFE (B). PRSs are unitless and expressed as relative values.

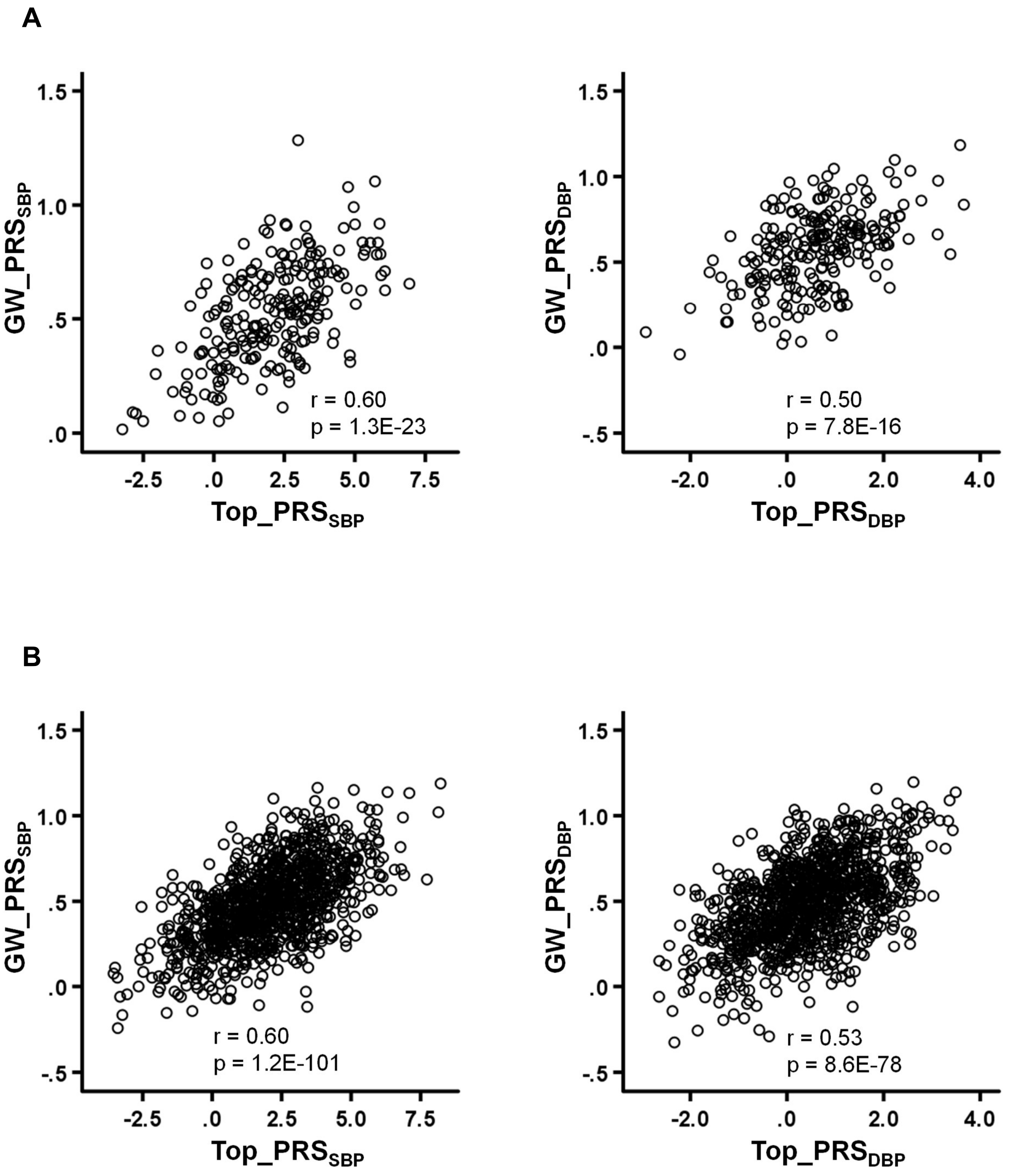

Supplementary Figure S3. Receiver operating characteristic (ROC) analysis for evaluation of genome-wide PRSs with blood pressure responses to hydrochlorothiazide.

The threshold for good response was set at covariate-adjusted BP change better than -0.5 s.d. (corresponding to -8.0 / -3.8 mmHg systolic/diastolic BP responses). The AUCs with 95% confidendece intervals are given. The optimal cut points for decision making, suggested by the highest Youden index values, are indicated by the arrows. The genome-wide PRSs are unitless and expressed as relative values. BP, blood pressure; AUC, area under curve.

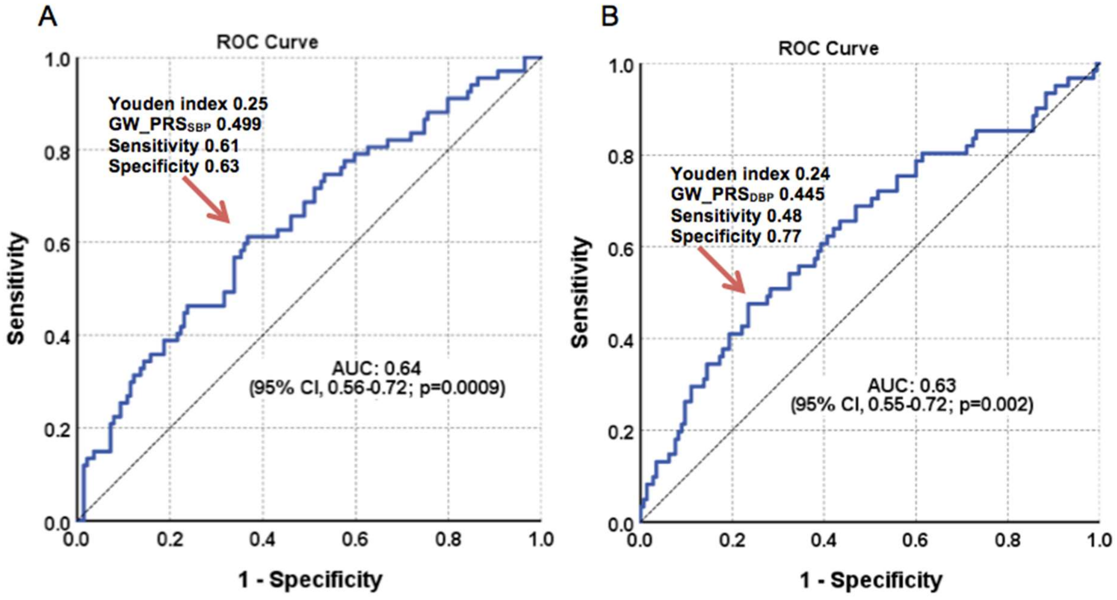

Supplementary Figure S4. Study protocols for GENRES and LIFE.
Arrows indicate occasions of blood pressure measurements. Ate, atenolol; HTZ, hydrochlorothiazide; Losa, losartan; Pre, prestudy period; Plac, placebo.

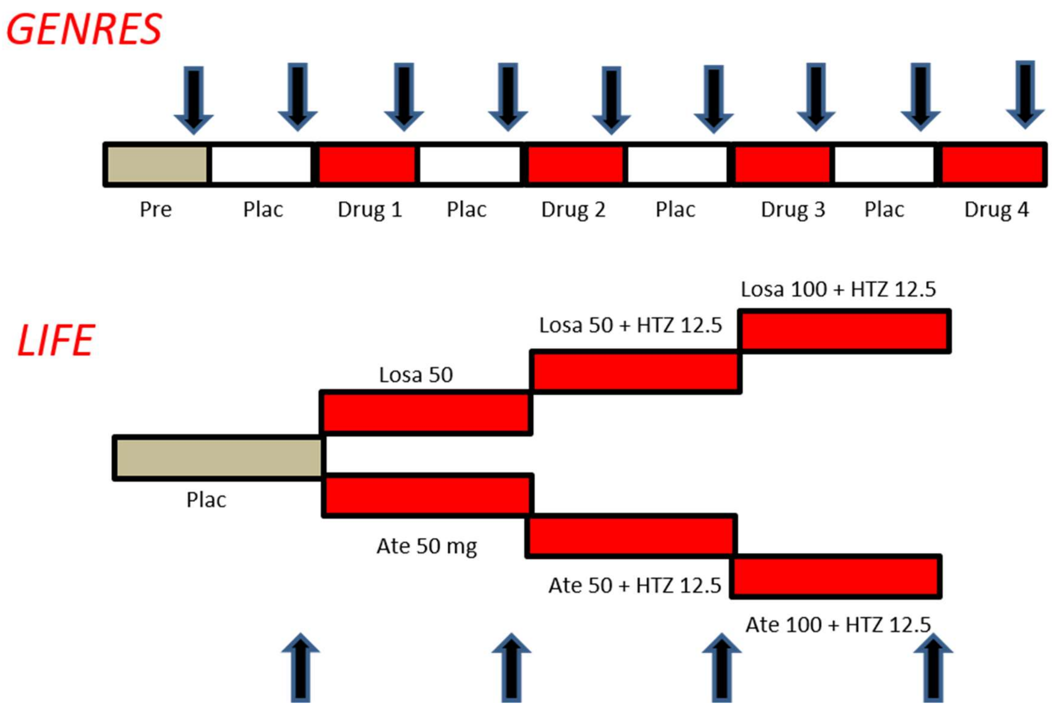

Supplementary Figure S5. Distributions of blood pressure residuals in GENRES.

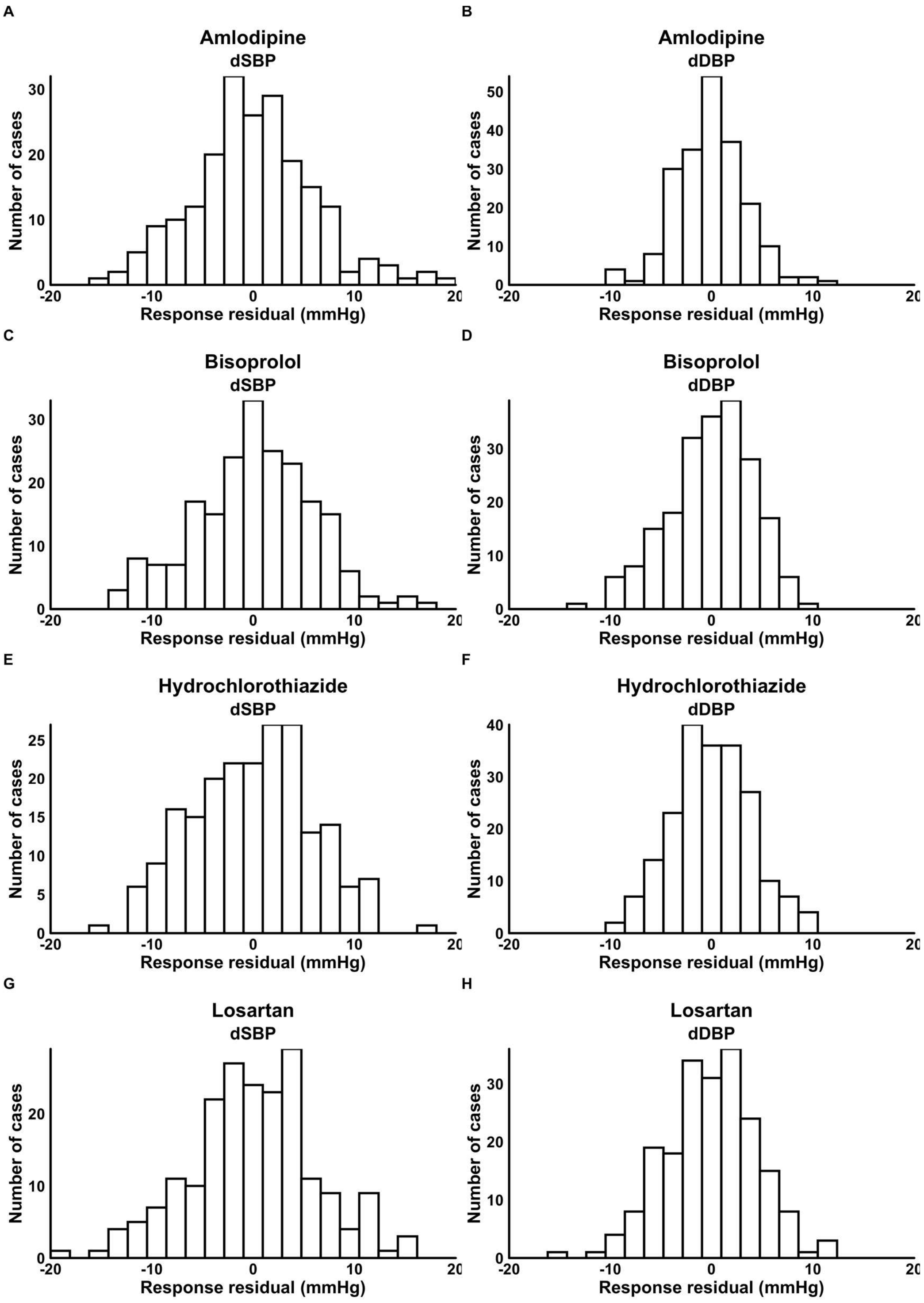

Supplement: Supplementary file 1 — Supplementary Information 1. [file 41598_2020_68878_MOESM1_ESM.pdf]
